# Experimental study on high-purity hydrogen generation from synthetic biogas in a 10 kW fixed-bed chemical looping system

**DOI:** 10.1039/c9ra03123e

**Published:** 2019-07-25

**Authors:** Sebastian Bock, Robert Zacharias, Viktor Hacker

**Affiliations:** Graz University of Technology, Institute of Chemical Engineering and Environmental Technology Inffeldgasse 25/C 8010 Graz Austria sebastian.bock@tugraz.at

## Abstract

The utilization of locally available renewable resources is crucial for the creation of a sustainable energy system in the future. Biogas, a product of the anaerobic digestion of biogenic residues, exhibits great potential as feedstock to generate hydrogen for fuel cell mobility applications. A 10 kW fixed-bed chemical looping research system, to-date the largest in the world, was operated to prove the applicability of this versatile process for synthetic biogas utilization. In this experimental study, the focus was laid on examining the influence of different operating parameters (biogas composition, steam co-feeding, process temperature) on the attainable hydrogen purity and system efficiency. The generated hydrogen, between 90 to 230 g per cycle, was characterized online by ppm-range gas analysis and exhibited a product gas quality between 99.8% and 99.998%. The difference observed is attributed to carbon deposition if synthetic biogas with an increased share of carbon dioxide was supplied. This study involved the longest uninterrupted period of operation of a lab prototype system for fixed-bed chemical looping with 250 hours of time-on-stream, four months of discontinuous service and 50 consecutive experimental cycles.

## Abstract 2

Experimental proof of synthetic biogas utilization for high-purity hydrogen generation (99.998%) with a 10 kW fixed-bed chemical looping system.

## Graphical abstract

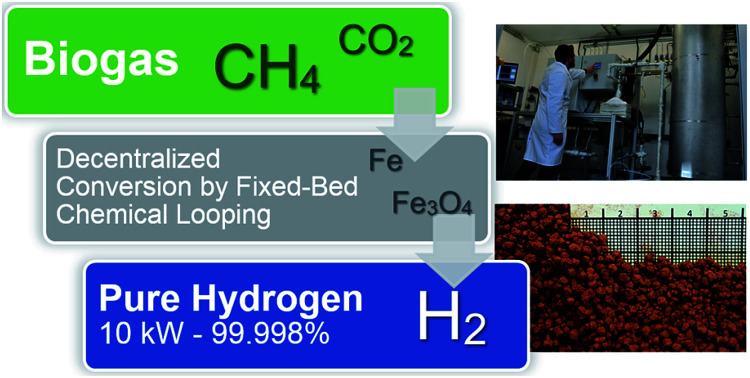

## Introduction

In the current discussion on the transition to a renewable energy system, hydrogen has been proposed as a suitable energy carrier and the missing link between fluctuating renewable sources and customer energy demands. Besides this, new local and regional energetic resources should be directly integrated into the energy system at the place of origin to avoid costly and energy-intensive transport. This is possible if technologies are used that are economically feasible in small scale and that can handle both fluctuating feedstock availability and energy demands.

Biogas is a product of the anaerobic degradation of organic material in landfills, wastewater treatment plants and digesters. It originates from a wide range of potential feedstock, depending on local available resources, such as various kinds of agricultural residues or energy crops.^1,2^ The produced biogas consists of methane (45–75%), carbon dioxide (25–45%) and other gases such as nitrogen (1–5%), as well as steam and oxygen in lower proportions.^3^ Moreover, a complex gas matrix of different trace gas compounds is present in biogas depending on the respective feedstock,^4,5^ which often hinders its direct utilization or severely affects downstream processes. Depending on the local opportunities available, biogas is currently utilized (i) for heat generation, *e.g.* for district heating, (ii) in CHP plants equipped with gas turbines or gas engines to co-generate both heat and electricity, (iii) upgraded to biomethane and co-fed into gas distribution networks or (iv) combusted with burners to prevent harmful CH_4_ emissions, *e.g.* in landfills.^6^ In 2014, 58.7 billion Nm^3^ of biogas were produced worldwide with an approximate energy content of 1.27 EJ (353 TW h), thereof about 50.5% in Europe.^7^ About 0.51 EJ (142 TW h) of heat and 0.29 EJ (80.1 TW h) of electrical power are generated worldwide from biogas.^7^

Several conventional conversion methods^8^ have already been proposed for the decentralized generation of pure hydrogen from biogas, such as steam reforming,^9^ dry reforming and autothermal reforming,^10,11^ but also unconventional methods, such as solar reforming, membrane reforming^12^ and thermal plasma reforming. However, these methods for hydrogen generation from biogas may be inhibited by the presence of common impurities as H_2_S which poisons downstream nickel-based catalysts, siloxanes or carbon depositions due to methane dry reforming with carbon dioxide.^8,13,14^

Despite its technical limitations, the decentralized generation of hydrogen is often not economically feasible especially in small scale due to the high costs for hydrogen generation, which range from 5.5 to 9 USD per kg H_2_.^9–11,15^ This is because low temperature fuel cells require very low impurity levels, demanding a pressure swing adsorption as additional purification step after steam reforming.

The Reformer Steam Iron Cycle (RESC), based on fixed-bed chemical looping and the steam iron process, is a promising method that can utilize different renewable resources to generate high-purity hydrogen. Its advantage is its simple process layout as proposed by Hacker *et al.*, which included a steam reformer and a steam-iron section within a single reactor, enabling concurrent hydrogen production and purification.^16,17^ Using this method allows a highly efficient hydrogen production with an efficiency up to 73%^18^ according to thermodynamic studies. Several studies have presented the feasibility of direct high-pressure hydrogen generation with a release pressure up to 100 bar in a fixed-bed chemical looping system, indicating purity levels of up to 99.97%.^19–21^

In recent years, an enlarged lab system was established to investigate the impact of upscaling this process from the level of small-scale test bench that involves several grams of oxygen carrier to a reactor system with a capacity of up to 20 kg of oxygen carrier combined with a reformer section for syngas generation.^22^

**Fig. 1:**
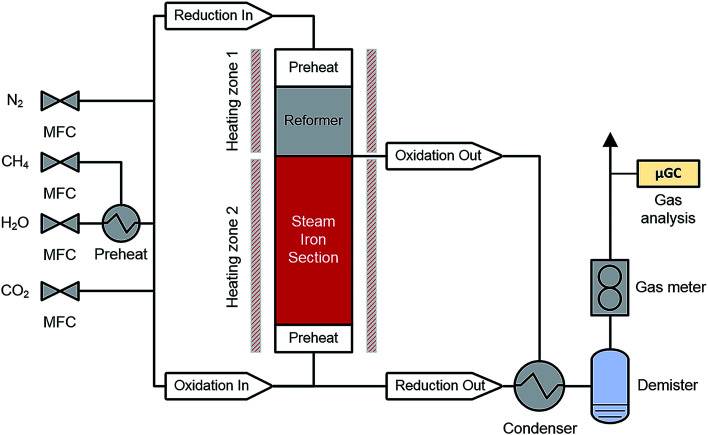
Schematic representation of the experimental setup of the fixed-bed reactor system.

A schematic representation of the RESC process is presented in Fig. 1. The synthetic biogas (considering CH_4_ and CO_2_ as main components) is converted into a synthesis gas by the dry reforming reaction (DR, eqn (1)) in the reformer section. However, in this experimental series steam is co-fed and methane is also converted by the steam methane reforming reaction (SMR, eqn (2)). The water-gas shift reaction occurs in parallel and leads to an equilibrium of CO, CO_2_, H_2_ and H_2_O in the synthesis gas (eqn (3)). In this study, an oxidative to reductive species ratio (O/R ratio), analog to the S/C ratio for steam reforming, was defined as the proportion of oxidative compounds (H_2_O and CO_2_) to reductive compounds (CH_4_) in the reforming reaction (eqn (4)).

1 CH_4_ + CO_2_ → 2CO + 2H_2_, Δ*H*_R,298_ = +205.9 kJ mol^−1^

2 CH_4_ + H_2_O → CO + 3H_2_, Δ*H*_R,298_ = +247.0 kJ mol^−1^

3 CO + H_2_O → CO_2_ + H_2_, Δ*H*_R,298_ = −41.1 kJ mol^−1^

4
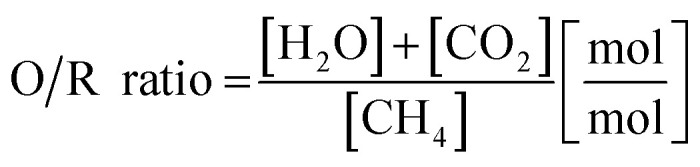


The reduction is carried out downstream the reformer section utilizing the generated syngas to reduce an iron-based oxygen carrier according to eqn (5) and (6). The oxidation of the reduced iron with steam in a subsequent process step enables the release of hydrogen by the reverse reactions of eqn (5) and (6). It is crucial to avoid solid carbon depositions during the reduction phase, since carbon is reoxidized during the oxidation phase. This causes the occurrence of carbon monoxide and carbon dioxide impurities in the hydrogen product gas. The underlying thermodynamic equilibrium is described by the Boudouard reaction and the direct methane decomposition (eqn (7) and (8)). Whereas the Boudouard reaction is favored at temperatures below 700 to 750 °C, the methane decomposition reaction is more likely at temperatures above 900–950 °C. To avoid harmful carbon deposition within commercial steam reformer systems, excess steam is co-fed to (i) lower the partial pressure of carbon monoxide and (ii) force the water-gas shift equilibrium towards carbon dioxide and hydrogen. However, a low O/R ratio is favorable to attain a highly reactive syngas for the reduction of iron oxides, although carbon formation is more likely. Therefore, it is crucial to determine an optimal operation point to ensure a high product gas quality by avoiding carbon depositions, but simultaneously ensuring a high degree of utilization of the feed gas.

5 Fe_3_O_4_ + H_2_/CO → 3FeO + H_2_O/CO_2_ Δ*H*_R,298_ = +74.7/+33.6 kJ mol^−1^

6 FeO + H_2_/CO → Fe + H_2_O/CO_2_ Δ*H*_R,298_ = +25.4/−15.7 kJ mol^−1^

7 CO → 0.5*C*_s_ + 0.5CO_2_, Δ*H*_R,298_ = −86.2 kJ mol^−1^

8 CH_4_ → *C*_s_ + 2H_2_, Δ*H*_R,298_ = 74.6 kJ mol^−1^

State-of-the-art investigations on the conversion of synthetic biogas to pure hydrogen with fixed-bed chemical looping systems are currently performed solely in micro-reactor systems, as presented by Lachen *et al.* and Plou *et al.*^23–26^ In these studies, different oxygen carrier materials were mixed with a nickel-based catalyst and tested in a test bench with an inner diameter of 13 mm. A single biogas mixture (50% CH_4_ : 50% CO_2_) was investigated and steam was co-fed to inhibit carbon deposition. Relatively low temperatures of 700 °C for reduction and 500 °C for oxidation were suggested by Lachen *et al.*^23–26^ to avoid particle sintering, however, this could inherently lower the reactivity of the gas–solid reaction. Lachen *et al.*^26^ reported impurities below the gas analytic system detection limit of 50 ppm for carbon monoxide in all cycles where steam was co-fed, but no explicit data for either carbon dioxide or methane contamination levels were provided to support the applicability of this method in low-temperature fuel cells.

Moreover, a high inert gas share of more than 50% was applied in the abovementioned studies. This share significantly influences the outcomes, as it decreases the probability of solid carbon deposition within the system by lowering the carbon monoxide partial pressure and, therefore, directly affects the Boudouard equilibrium. In industrial applications for biogas upgrading, a high inert gas share may be inefficient due the high levels of inert gas consumption required for system operation.

Another study was presented by Galvita *et al.*, who investigated the applicability of biogas mixtures in a catalyst-assisted, fixed-bed chemical looping system without steam addition in a micro-reactor with an inner diameter of 10 mm.^27^ The system had an output of 3.5 mol H_2_ per kg Fe_2_O_3_. The hydrogen purity was not explicitly specified for the investigated operating points, but a range between 100 and 200 ppm was given in the study (99.98% to 99.99%).

An earlier experimental series was also performed by Nestl *et al.* in a smaller lab reactor system with a combined reformer and steam iron section,^28^ comprising of 300 g of oxygen carrier inventory and an inner diameter of 35 mm. The attained hydrogen purity in two experiments feeding a biogas mixture with steam addition was 99.75% and 99.84%, respectively.

However, the influence of endothermic and exothermic reactions in all of these reactor systems with diameters between 10 and 35 mm is considerably lower compared to larger fixed-bed reactors as necessary for industrial applications. In particular, the presented 10 kW lab reactor system is unique because it is considerably larger with an oxygen carrier inventory of 18 kg and an inner diameter of 124 mm.^22^ Thus, it can be used to proof the applicability of different process mechanisms and feedstocks for potential applications.

The goal of this study was to experimentally verify and define certain limitations of typical biogas compositions with respect to their application in a 10 kW fixed-bed chemical looping system. Two synthetic biogas compositions with different O/R ratios were selected on the basis of previous study findings with steam methane reforming.^22^ To address the limitations that were observed during previous studies conducted on smaller test benches, a lower partial pressure of nitrogen (used as internal standard) was applied to ensure minimal effects on the thermodynamic equilibria. The impacts of the process temperature, different biogas compositions and steam addition on feedstock utilization and hydrogen purity were determined. The experimental study was performed in the largest 10 kW fixed-bed chemical looping research system currently available worldwide.

## Experimental

### Materials

The applied pelletized oxygen carrier material consisted of 80% Fe_2_O_3_ and 20% Al_2_O_3_ powder (20–60 μm, both Alfa Aesar). The powders were dry-mixed and pelletized with water as a binding agent using an intensive mixer (Eirich EL1). The pellets were dried at 150 °C and calcined at 900 °C for six hours. A commercially available catalyst for steam reforming applications was used for the reformer. The preheating zone for the gaseous feed streams in the reactor system consisted of Al_2_O_3_ pellets (3.18 mm, Alfa Aesar).

### Experimental setup

The experiments were carried out in a fixed-bed reactor system (*d*_i_ = 124 mm, *L* = 1800 mm, see Fig. 1). The reactor consisted of two parts, (i) the reformer section and (ii) the steam iron section. The reformer section comprised a preheating bed for the feed gas and the reformer catalyst (4.8 L). The steam iron section comprised the oxygen carrier material (18 kg) and a preheating bed for the feed gas. The reactor was heated with an electric furnace, whereby the temperatures of both sections could be separately controlled. Thermocouples were introduced through side tubes to measure the temperature distribution inside the reactor system. To avoid carbon formation in cold zones, nitrogen was applied as purging gas (*V*_N_2_, tot_ = 5 NL min^−1^) to these side tubes.

All gaseous components (CO_2_, N_2_ and CH_4_) were supplied with mass flow controllers (Bronkhorst High-Tech). Steam was fed using a direct evaporator system (ADrop DV-3) with a liquid flow controller (Bronkhorst High-Tech), mixed with methane and heated to 200 °C in an integrated superheater. The feed gas entered the reactor with a temperature of 150 °C.

The outlet gas was cooled in a condenser unit and conditioned with a demister. The product gas was continuously analyzed with a micro-GC (Inficon Fusion) with a 10 m mole sieve column, a 12 m Pora-Plot-Q column and a separate mole sieve module for ppm-range detection of CO and CH_4_. The detection limit for the carbonaceous compounds CO_2_, CO and CH_4_ was 3 ppm.

Each experiment (cycle) consisted of two phases:

(i) The reduction phase, during which the feed gas was supplied to the reformer section and converted to a syngas. The iron oxide was subsequently reduced in the downstream steam iron section. All reduction phases were carried out for three hours unless otherwise stated.

(ii) The oxidation phase, during which steam (66.7 g min^−1^) was directly introduced into the steam iron section and oxidized the iron-based oxygen carrier, resulting in the formation of hydrogen. The reformer section was bypassed to minimize the contamination of the gas by reoxidation of solid carbon in the reformer. Reoxidation was carried out until complete oxygen carrier conversion was achieved.

The reactor wall heating temperature in the reformer section was set to 900 °C. In the steam iron section, two temperature levels of 750 °C and 850 °C were investigated. During both reaction stages, 10 NL min^−1^ nitrogen were supplied as an internal standard along with the feed gas (5 NL min^−1^) and fed in through the side pipes (5 NL min^−1^). The reactor was purged with nitrogen (10 NL min^−1^) between the reduction and oxidation phase to exclude reduction gas remains from the product gas in oxidation phase.

On the basis of data obtained from the literature for biogas applications, two different synthetic biogas compositions (CH_4_ : CO_2_ = 75 : 25% and 45 : 55%) were chosen as feed for the reduction phase. With regard to the recent studies conducted by our research group, a steam to carbon ratio (S/C ratio) of 1.2 and 1.6 have been observed to provide optimal process efficiency while simultaneously avoiding harmful carbon deposition.^22^ To attain a comparable O/R ratio during this experimental series, steam was co-fed with the synthetic biogas. In addition, a reference operation point was defined with solely steam methane reforming at a S/C ratio of 1.2, that yielded a high process efficiency and purity in a previous experimental series (Table 1).^22^

**Table 1:** Operating points of the experimental series

Operating point	CH_4_ : CO_2_	O/R
REF	—	1.2
1	75 : 25%	1.2
2	45 : 55%	1.2
3	75 : 25%	1.6
4	45 : 55%	1.6

Catalyst testing and determination of the reformer gas composition was performed bypassing the steam iron section. Every operating point was analyzed until a stable gas composition in the off-gas and a steady temperature distribution in the reformer were reached.

Two process parameters characterize the steam iron process with regard to the Baur–Glaessner diagram:^16^ the steam ratio and the carbon dioxide ratio (see eqn (9) and (10)). The amounts of hydrogen, carbon monoxide and carbon dioxide were determined from the dry gas composition. Therefore, the carbon dioxide ratio was directly determined using the gas analytic system, whereas the amount of steam for the steam ratio was calculated from the hydrogen mole balance and, therefore, was more inaccurate.9
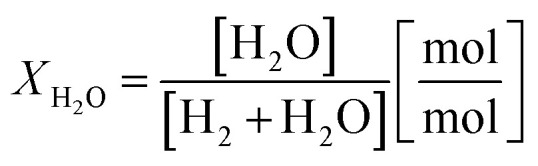
10
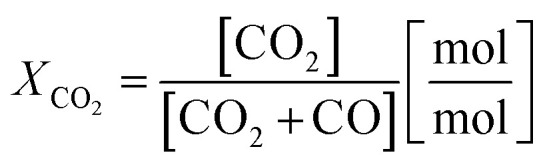


A calculation of the thermodynamic equilibrium for the reformer gas composition was carried out with ASPEN Plus v8.4 with Peng–Robinson EOS by minimizing the Gibbs free energy.

## Results and discussion

### Reformer characterization

**Fig. 2:**
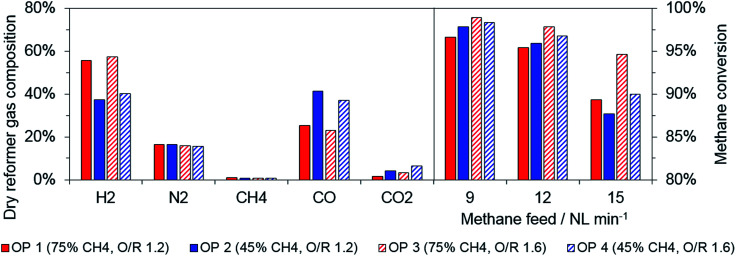
Reformer gas composition for all biogas operation points (12 NL min^−1^ feed flow, left) and methane conversion in reformer for different feed flow (right).

First, the reformer gas was characterized to identify certain limitations for the experimental series, as the applied catalyst has been proposed primarily for steam reforming applications. The dry gas composition, as shown in Fig. 2, primarily depends on the biogas composition. The hydrogen amount increases as the share of methane in the biogas increases, hence, MSR is favored if a higher share of steam is added to the feed gas to attain the desired O/R ratio. Accordingly, a higher carbon monoxide share was measured for biogas as the amount of carbon dioxide increases, because mainly MDR is present. These data were in accordance with those obtained from thermodynamic equilibrium simulations from ASPEN Plus, which were performed to validate the experimentally acquired data.

As expected, an increasing amount of unconverted methane was determined for higher feed gas flow for all operation points (see Fig. 2, right). In general, a higher O/R ratio increased the methane conversion rate at all operating points. Hence, the relative share of methane in the dry reformer gas was below 1% for a methane conversion above 95%. With respect to the results, the methane feed in the reduction phase was fixed to 12 NL min^−1^ for all operation points. The absolute methane stream was defined as constant to maintain a consistent feeding rate of the active reductive gas component (see Table 2).

**Table 2:** Reduction phase operating parameters for all operating points

Operating point	Parameters		Reduction feed gas flow		
	CH_4_ : CO_2_	O/R	CH_4_ NL min^−1^	CO_2_ NL min^−1^	H_2_O g min^−1^
1	75 : 25%	1.2	12	4	8.4
2	45 : 55%			14.4	0
3	75 : 25%	1.6		4	12.2
4	45 : 55%			14.4	3.9
REF	—	1.2		0	11.6

### General reduction and oxidation properties

**Fig. 3:**
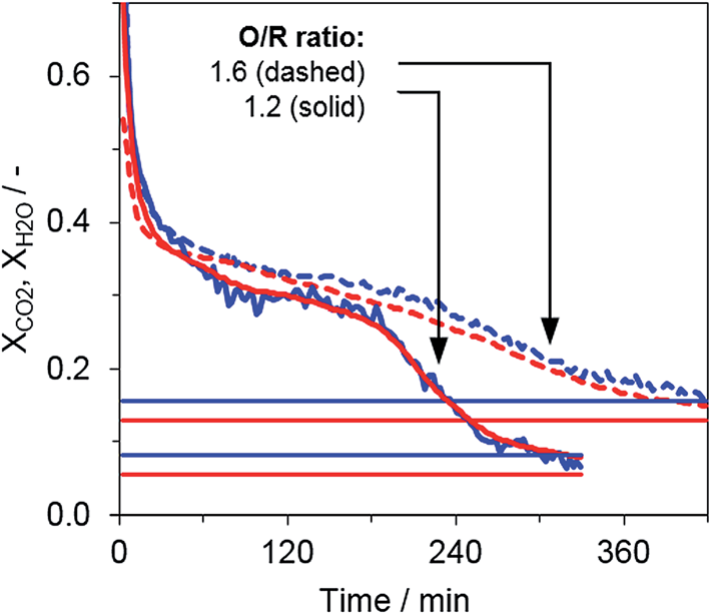
Steam ratio *X*_H_2_O_ (blue) and carbon dioxide ratio *X*_CO_2__ (red) for the full reduction with synthetic biogas (75% CH_4_, 25% CO_2_). Horizontal lines represent the respective reformer gas composition from previous reformer tests.

Two consecutive cycles illustrate the general reduction behavior in the system for two operation points with different O/R ratios, 1.2 (OP 1) and 1.6 (OP 3), but a constant biogas composition (75% CH_4_, 25% CO_2_). The results for the steam ratio *X*_H_2_O_ and carbon dioxide ratio *X*_CO_2__, as indicators for the reduction progress, displayed the characteristic behavior of fixed-bed steam iron processes (see Fig. 3). The reduction phase can be divided in two subsections, (i) where Fe_3_O_4_ is reduced to FeO and Fe with a higher gas conversion observed in the beginning (up until approx. minute 50) and (ii) where Fe_3_O_4_ is consumed and a steady steam- and carbon dioxide ratio between 0.3 and 0.4 is reached according to the gas–solid equilibrium.^16^ At the end of the respective cycle, the syngas conversion declined because lower amounts of FeO were present in the oxygen carrier bed and ended close to the measured reformer gas composition. Throughout the whole experimental series, the Fe_3_O_4_–FeO gas equilibrium with a steam- and carbon dioxide ratio of about 0.7 was not clearly visible, a result that is in accordance with those of several other experimental analyses.

The reduction reaction showed a reaction front similar to that presented in a previous publication,^22^ but the temperature in the center of the oxygen carrier bed was lowered by only 10 to 15 °C *via* the endothermic reduction, due to the low reducing gas stream and the very broad characteristic of the reaction front. As shown in Fig. 3, the full reduction of the iron oxide material was considerably slower when higher excess oxidative compounds were present in the feed gas (O/R ratio of 1.6 *vs.* 1.2), although the same absolute feed gas flow of methane was applied. Therefore, the results indicate that a higher O/R ratio, in turn, impedes the degree of utilization of methane as valuable feed.

During the oxidation phase, the hydrogen product gas flow immediately rose to a stable conversion of about 65%, exhibiting a good correlation to the gas–solid equilibrium of iron and wuestite (FeO). Again, a period of declining conversion was observed at the end of the oxidation phase.

**Fig. 4:**
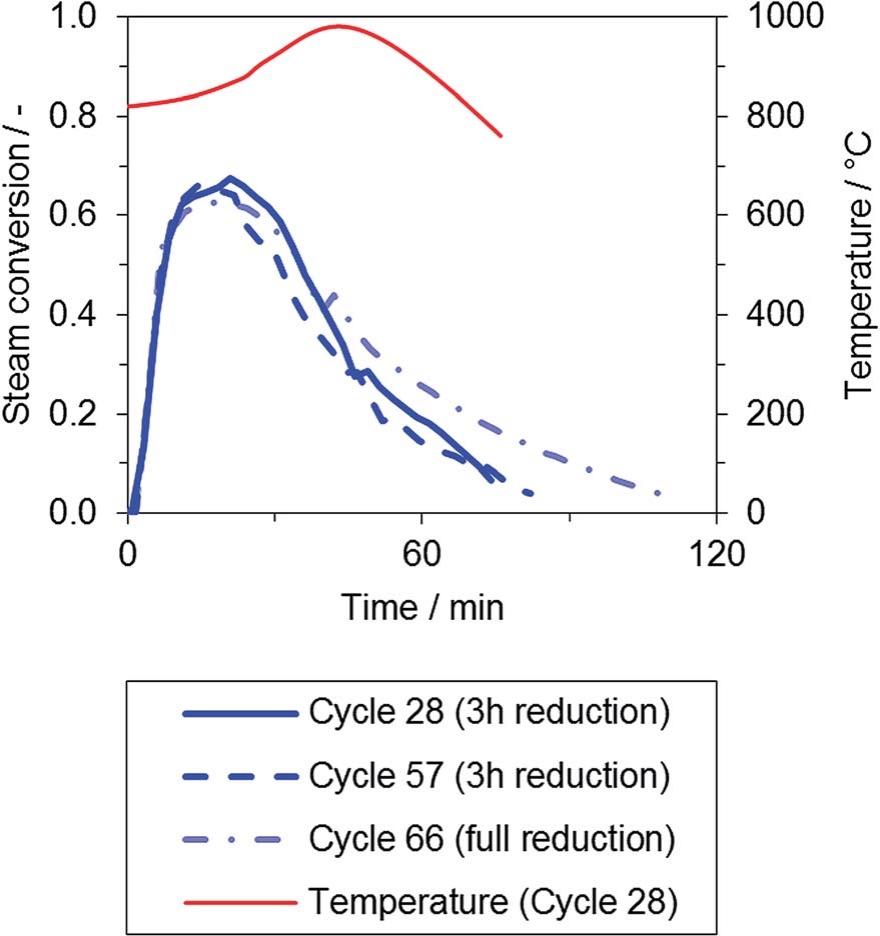
Representative steam conversion during the oxidation phase after reduction with synthetic biogas (OP 1, 75% CH_4_, 25% CO_2_, O/R ratio = 1.2, blue) and temperature in the center of the oxygen carrier bed (red).

The passing reaction front also clearly indicates the oxidation progress in the reactor system, as presented in Fig. 4: the temperature *T*_axis_ (red) is measured near the end of the oxygen carrier bed. Due to the exothermic oxidation reaction, it was noted that the temperature in the center of the oxygen carrier bed is significantly affected in large-scale fixed-bed applications. The temperature increased to a maximum between 940 and 1000 °C, and a high self-heating rate that ranged from 1.7 to 4.8 °C min^−1^, averaged from temperature rise onset to offset, was determined throughout the experimental series. Both effects were dependent upon the degree of reduction in the respective cycle. However, these effects may significantly influence the oxygen carrier integrity, an effect that should be considered when characterizing materials applied in fixed-bed operation.

The material degradation observed throughout the whole test series (50 cycles, one cycle including reduction and oxidation phases) is characterized by the decline in the oxygen exchange capacity, which is defined by the amount of hydrogen produced after a full reduction of the oxygen carrier. Based on the available results, no significant loss of the oxygen exchange capacity was observed throughout the experimental series.

### Effect of biogas composition

**Fig. 5:**
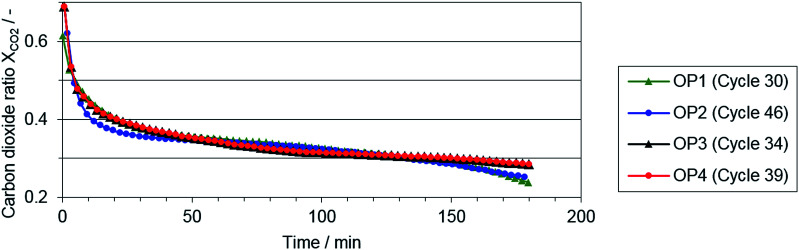
Representative carbon dioxide ratio *X*_CO_2__ in the reduction phase at operating points 1–4.

In Fig. 5, the carbon dioxide ratio is shown, serving as an indicator for the reaction progress of the reduction phase. Only small deviations between the single operating points are visible, although the applied syngas from the reformer varied significantly at the single operating points (see reformer characterization). Nevertheless, at the end of both reduction phase with a lower O/R ratio (OP 1 and 2), a lower carbon dioxide ratio indicates a higher reduction progress.

**Table 3:** Amount of hydrogen produced in oxidation phase at different operating points (average value of three cycles)

O/R ratio	Biogas composition: CH_4_ : CO_2_ ratio	
	75 : 25%	45 : 55%
1.2	OP 1	OP 2
	2465 NL/100%	2240 NL/91%
1.6	OP 3	OP 4
	2050 NL/83%	1850 NL/75%

This aspect of higher reduction progress at lower O/R ratios is also revealed by the hydrogen output in the oxidation phase (see Table 3). With regard to the results, both an increasing O/R ratio and carbon dioxide share in the feed gas lowered the quantity of hydrogen as product gas in the oxidation phase. Comparing the amount of hydrogen produced at the reference operating point (SMR, S/C ratio 1.2) with the most comparable operating point 1 (biogas with 75% CH_4_ : 25% CO_2_, O/C ratio 1.2), no significant difference was found in later experiments (see Table 4).

Furthermore, the fixed-bed process also enables the storage of chemically bonded energy for hydrogen generation in the form of reduced iron by deferring the hydrogen discharge and storing the reduced oxygen carrier over long periods. At least six consecutive cycles were performed for every operating point, (i) three with overnight storage (15 to 16 hours) of the reduced oxygen carrier and concurrent purging of the reactor and (ii) three with immediate hydrogen release after 15 minutes of purging.

**Fig. 6:**
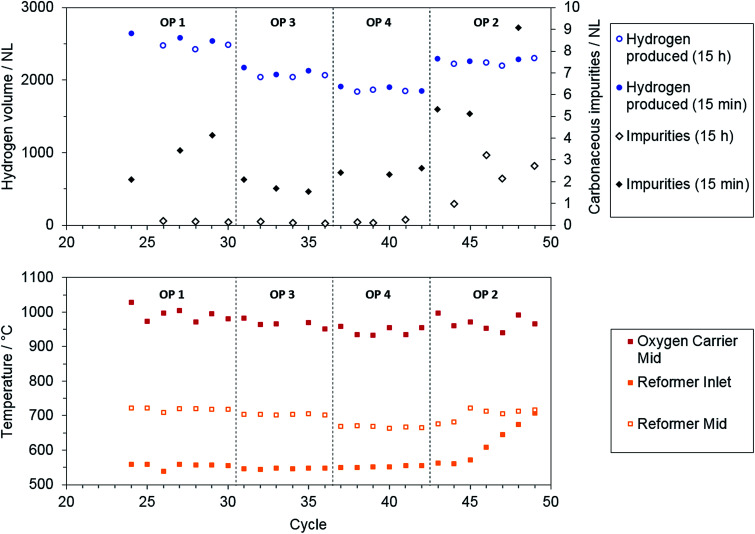
Results of repeated cycling at operating points 1–4: hydrogen output and total amount of carbonaceous impurities for overnight purging (empty) and 15 minutes purging (filled) (top) and characteristic temperatures on the reactor axis (bottom).

As Fig. 6 shows, the produced amount of hydrogen was comparable for the direct release of hydrogen after 15 minutes and for overnight storage at all operating points. The maximum temperature reached in the oxidation cycle, as measured by a thermocouple situated in the center of the oxygen carrier bed, also showed no significant trend between the single operation points. A major difference was found for carbonaceous impurities during the oxidation phase. The elongated purging (non-filled circles and squares) significantly improved the attainable hydrogen purity. This finding is attributed to incomplete purging of the reactor system from carbonaceous gases from reduction phase after a purging duration of 15 minutes.

Considering the hydrogen purity obtained when the system was purged overnight, the total carbonaceous impurities were below 0.3 NL for all operating points with steam addition in reduction phase (OP 1, 3, 4). Only operating point 2 exhibited significantly higher carbon impurities in the product gas. In addition, the total amount of impurities still increased over several cycles for both purging methods (see Fig. 6, OP 2). From these results, it can be assumed that severe carbon deposition was present only at operating point 2 with solely dry reforming of the methane feed and a low O/R ratio of 1.2.

To recover the steam iron section from carbon deposition after the experimental series, steam was applied in the same manner as in oxidation phase after cycles 47 and 49 to reoxidize solid carbon. A maximum of 0.35% and 0.45% of carbon-containing impurities, respectively, were found in the off-gas with a co-fed inert purging gas for cycles 47 and 49. This recovery of the steam iron section had only minor effects on the hydrogen purity in subsequent cycles (see impurities for cycles 48 and 49 in Fig. 6).

A more likely reason that the hydrogen purity declined was found in the reformer section. A significant degradation of the reformer catalyst was ascertained as the minimum reformer inlet temperature increased constantly at operating point 2 (see Fig. 6). By repeating the reformer characterization after the experimental series this result was confirmed, as the methane amount in the dry reformer syngas increased from 0.6 to 1.4%.

A subsequent steam oxidation step of the reformer section, bypassing the steam iron section, led to a maximum of 14% carbon-containing gases in the co-fed purging gas. This ratio was considerably higher than observed during the oxidation of solid carbon in the steam iron section presented before. That said, the contamination of the hydrogen product gas may have been caused by unintended oxidation of solid carbon depositions in the reformer section during the oxidation phase caused by the combined reactor unit. Moreover, the initial activity of the reformer could not be completely restored by steam oxidation, since the temperature at the reformer inlet was still significantly higher (680–710 °C) in later experiments, including all operating points, than it had been before.

### Variation of process temperature

In this the experimental series, the process temperature of 850 °C was chosen on the basis of previous experimental findings. This temperature level typically exhibits (i) beneficial thermodynamics and reaction kinetics for iron-oxide reduction, (ii) lower probability for carbon depositions from Boudouard equilibrium favored at temperatures below 700 to 750 °C and (iii) nearly complete methane conversion for steam reforming with above 800 °C. Nonetheless, lower process temperatures have been proposed by other research groups, as they mitigate challenges faced in the selection of materials for fixed-bed chemical looping. They are also beneficial in terms of heat integration due to lower heat losses and a more efficient use of high-temperature heat in industrial applications.

A comparative experimental series was performed at temperatures of 750 °C and 850 °C in the steam iron section. All other parameters, including the reformer temperature, were kept constant to provide unaltered boundary conditions for the experimental series. In order to minimize the impact of oxygen carrier degradation on the total amount of hydrogen produced, only one cycle was performed per operation point.

In Table 4, the results for the produced amount of hydrogen during the oxidation phase are shown for a fixed reduction duration of three hours. The produced amount of hydrogen declined significantly at all operation points for the lower temperature of 750 °C. However, operating points with a higher share of CO_2_ in the biogas were less strongly affected.

**Table 4:** Hydrogen produced for different operating points at 850 °C and 750 °C

O/R ratio	Temperature/°C	Biogas composition: CH_4_ : CO_2_ ratio		
		100 : 0	75 : 25	45 : 55
1.2		REF	OP 1	OP 2
	850	2260	2260	2080
	750	1390	1490	1590
	**Difference**	**−39%**	**−34%**	**−24%**
1.6		—	OP 3	OP 4
	850	—	1910	1660
	750	—	820	1100
	**Difference**	**—**	**−57%**	**−33%**

The reason is explained with the Baur–Glaessner diagram: the gas equilibrium for the conversion of wuestite to iron between 850 °C and 750 °C increases for the carbon dioxide ratio, but decreases for the steam ratio. Overall, lowering the process temperature may have other advantages, but significantly lowers the feedstock utilization.

### High-purity hydrogen discharge

The application of chemical looping technologies for high-purity hydrogen production places a focus on solid carbon deposition, as they are reoxidized by steam during the oxidation phase and contaminate the product gas. As low-temperature fuel cells require low amounts of contaminants, the attainable hydrogen purity was investigated. The ISO 14687-2 defines the maximum tolerable hydrogen impurities for road vehicles to below 2 ppm of total hydrocarbons (methane basis), 2 ppm of carbon dioxide and 0.2 ppm for carbon monoxide.^29^

Between the reduction and oxidation phases, the reactor system was purged overnight (15 to 16 hours) to exclude gaseous residues from the reduction phase. Nevertheless, residues of carbon monoxide and carbon dioxide were still present in the purging gas to a total extent of 0.3 N mL min^−1^ (typ.) for OP 1, 2 and 4 and 2 N mL min^−1^ (typ.) for OP 4. To evaluate the hydrogen purity, the first and last 5% of hydrogen produced were excluded from the calculation, because a previous experimental series indicated that the highest relative impurities occur at the beginning and end of the oxidation phase.^22^

**Fig. 7:**
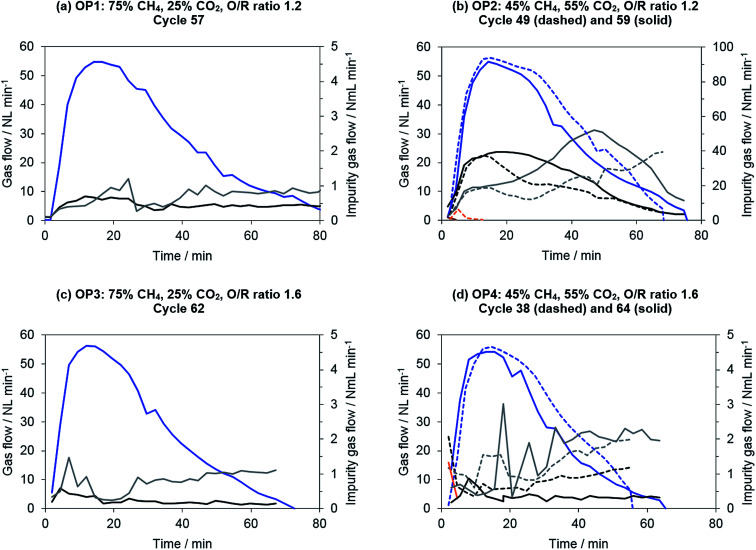
Representative product gas composition in oxidation phase with hydrogen (blue), carbon monoxide (black), carbon dioxide (grey) and methane (orange). To represent the unsteady behavior, two experimental results are included in (b) and (d). Note the different scales along the secondary *y*-axis for operating point 2 (upper right).

The results of the product gas purity are shown in Fig. 7. Operating points with lower shares of carbon dioxide in the biogas (75% CH_4_, 25% CO_2_) exhibited an almost constant level of impurities of below 1 N mL min^−1^ per component. This result was reproducible throughout the whole experimental series, so that no severe carbon deposition can be assumed. The product gas purity of the best 90% of hydrogen obtained ranged from 20 to 40 ppm. Moreover, the reference operating point (SMR, S/C ratio 1.2) exhibited similar results, with a total hydrogen purity of 35 ppm of total impurities obtained. This means that no distinct differences can be found between the appliance of synthetic biogas and methane for distinct operating points. Furthermore, the results for steam–methane reforming (reference operating point) are comparable to those reported in a previous publication presented by our research group.

Operating points with a higher share of carbon dioxide in the biogas (OP 2 and OP 4: 45% CH_4_, 55% CO_2_) exhibited unsteady levels of impurities (see Fig. 7). At a higher O/R ratio of 1.6 (OP 4), the impurities were more irregular, although they were still at a low level of about 2 N mL min^−1^ per component. The hydrogen purity attained was still between 40 to 60 ppm related to the total amount of hydrogen produced.

Severe contaminations were observed only at operating point 2 with solely dry reforming at an O/R ratio of 1.2. The measured carbonaceous impurities were more irregular in each of the single oxidation phases, and the total flow of contaminants was significantly higher (20 to 50 N mL min^−1^) compared to all other operating points. The total amount of impurities in the product gas increased consequently to between 1000 and 1500 ppm.

For all operating points with low carbonaceous impurities (OP 1, 3, 4), carbon monoxide accounted for about 20 to 30% of the impurities and carbon dioxide for about 70 to 80%. No methane was found in the product gas feed if the system had been sufficiently purged of residues from the reduction gases. An increase of the absolute carbonaceous species flow during oxidation phase (see Fig. 7) can be explained by the experimental layout. Carbon deposition induced by the Boudouard equilibrium (eqn (7)) is favorable at a high partial pressure of carbon monoxide. The highest partial pressure of carbon monoxide occurred at the reformer outlet respectively the chemical looping inlet. This results in an increased probability of carbon formation in this area. However, in the subsequent oxidation phase, this section becomes the hydrogen outlet. As the steam conversion decreases with ongoing conversion of the oxygen carrier, the steam partial pressure at the oxidation outlet increases. Therefore, a higher oxidation rate of deposited carbon is likely at the end of the oxidation phase, leading to a higher absolute amount of trace gas impurities.

To determine the influence of the process temperature, high-purity experiments were also performed at 750 °C with elongated purging. The absolute amounts of impurities at all operating points at 750 °C were comparable to those observed for experiments performed at 850 °C. However, because the total amount of hydrogen produced at 750 °C was lower, the relative amount of carbonaceous contaminants increased to between 50 and 70 ppm (OP 1, 3, 4) and 2311 ppm (OP 2). Therefore, no significant benefit in terms of process efficiency or attainable hydrogen purity was found by lowering the process temperature of the reduction and oxidation phase simultaneously.

## Conclusions

A fixed-bed chemical looping system was operated to proof the ability of high-purity hydrogen generation for low temperature fuel cells with synthetic biogas. The commercial steam reforming catalyst was suitable for biogas reforming as long as a sufficient proportion of steam was co-fed. Severe degradation was caused by solely dry reforming and led to a non-regenerable decrease in catalytic activity within 21 hours of operation.

The applicability of different biogas compositions was investigated by co-feeding steam to attain a desired oxidative to reductive species ratio (O/R ratio). The hydrogen production at an O/R ratio of 1.6 was 15–20% lower than that at an O/R ratio of 1.2. Likewise, a higher share of dry reforming reduced the hydrogen output based on an identical amount of methane supplied.

The attainable hydrogen purity was largely affected by (i) the purging of the reactor system prior the steam oxidation and (ii) the prevention of solid carbon depositions during the reduction phase. The total carbonaceous impurities for applicable operating points were between 20 and 40 ppm with a proportion of 20% carbon monoxide and 80% carbon dioxide. A slight addition of steam was always necessary to prevent severe solid carbon deposition in the reduction phase.

A lower process temperature had considerably disadvantageous influence on both the process efficiency and hydrogen purity: The amount of produced hydrogen, after a fixed reduction time of three hours, decreased by 24–57% for a process temperature of 750 °C compared to 850 °C. Since the absolute amount of impurities remained almost constant for both temperature levels, the relative amount of impurities in the product gas increased by 50–100% at 750 °C.

## Conflicts of interest

There are no conflicts to declare.

## Supplementary Material
